# Editorial: Endocrine regulation and physiological adaptation of stress response in aquatic organisms

**DOI:** 10.3389/fphys.2024.1406986

**Published:** 2024-04-08

**Authors:** Yiming Li, Yi-Feng Li, Marco António Campinho, Juan Fuentes

**Affiliations:** ^1^ Fishery Machinery and Instrument Research Institute, Chinese Academy of Fisheries Sciences, Shanghai, China; ^2^ International Research Centre for Marine Biosciences, Ministry of Science and Technology, Shanghai Ocean University, Shanghai, China; ^3^ Key Laboratory of Exploration and Utilization of Aquatic Genetic Resources, Ministry of Education, Shanghai Ocean University, Shanghai, China; ^4^ Algarve Biomedical Centre-Research Institute, Faro, Portugal; ^5^ Faculty of Medicine and Biomedical Sciences, University of the Algarve, Faro, Portugal; ^6^ Instituto de Ciencias Marinas de Andalucía (ICMAN), Consejo Superior de Investigaciones Científicas (CSIC), Puerto Real, Spain

**Keywords:** stress response, aquatic organisms, endocrine regulation, physiological adaptation, homeostasis

Organismal growth is a complex, genetically regulated process that integrates various physiological signaling pathways, where endocrine regulation is pivotal. In fully developed animals, endocrine regulation plays a central role in maintaining homeostasis and adapting to changing environmental and biological conditions. In aquatic organisms, environmental stressors such as environmental temperature, hypoxia, salinity changes, and exposure to pollutants can disrupt homeostasis, leading to physiological, molecular, and behavioral responses. Understanding the molecular and cellular mechanisms of endocrine regulation and physiological adaptation in response to environmental stresses is crucial, significantly impacting aquatic ecosystems. The main objective of this Research Topic was to explore and discuss these mechanisms, providing valuable insights into aquatic animal biology and adaptation.

Several studies have provided valuable insights into various fish and mollusk species’ physiology and adaptation mechanisms in response to environmental stressors.

Ruiz et al. explored the physiological responses of rainbow trout under repeated hypoxia exposure, revealing resistance patterns to anoxic events.

Ma et al. investigated the utilization of carbohydrates in fish after intraperitoneal injection or oral administration of glucose or fructose, highlighting the effects on blood sugar levels and hypoxia tolerance. In addition, turbot and tiger fish are intolerant to acute hypoxia, and adding glucose or fructose improves hypoxia tolerance in both marine fish species by activating anaerobic glycolysis. The study provides essential scientific information for understanding the mechanism of glucose and fructose utilization in fish and improving hypoxia tolerance.

Cheng et al. delved into the molecular regulation mechanisms of high-temperature environments in paddy field carp (PF-carp), shedding light on biochemical parameters and gene pathways affected by heat stress.

Wang et al. described the transcriptional temperature response on *Bombyx Mori* larva. These results contribute to a further understanding of the mechanism of high-temperature resistance in invertebrates in the context of global warming. Qiu et al. identified that McNrf2 could protect mussels from benzopyrene-induced oxidative stress (Bap) by inhibiting McSLC35E2. They further describe potential *McNrf2* target genes after ChIP-seq, revealing the highly complex regulation responses to oxidative stress in marine invertebrates.

Liu et al. studied the toxic effect of ammonia nitrogen stress on the intestinal tract of banded catfish (*Pelteobagrus fulvidraco*). The findings of this study suggest that ammonia nitrogen stress destroys the intestinal mucosal barrier and induces intestinal inflammation, which provides valuable data for intestinal immunotoxicology studies in aquatic organisms.

Holhorea et al. combined biometric, behavioral, physiological, and external tissue damage scoring systems to understand the endocrine response to different stocking densities. This suggests that the growth-regulatory shift in high-density fish cultures supports active rather than passive behavior, which is thought to be adaptive and can maintain active and synchronized feeding behavior while minimizing the risk of oxidative stress and epidermal skin damage.

Zeng et al. identified the *elovl8* gene from *P. fulvidraco* and analyzed its evolutionary and molecular characteristics and transcriptional changes under different nutritional states. This analysis indicates that *elovl8* is involved in HUFAs biosynthesis in early development.

Impellitteri et al. investigated the response of mediterranean mussel (*Mytilus galloprovincialis*) to chlorpromazine (Cpz). These results indicate that Cpz can cause non-specific biochemical and cellular disorders even at low picomolar concentrations, which is significant for healthy culture and ecotoxicity studies of purple mussels.

Zhang et al. investigated the effects of microplastics on intestinal morphology and inflammatory response of Largemouth bass (*Micropterus salmoides*) (carnivorous fish), grass carp (*Ctenopharyngodon idella*) (herbivorous fish), and swordfish (*Xiphias gladius*) (omnivorous fish) with different feeding methods. Different fish’s genetic responses differed according to different sizes and concentrations of microplastic exposure. The reasons for the different effects of microplastics on fish are unknown but may be due to differences in the structure and function of the digestive system. The results of this study provide a theoretical basis for further analysis of the pathological mechanism of fish intestines caused by microplastics.

Liu et al. used golden cuttlefish (*Sepia esculenta*) and ricefield eel (*Monopterus albus*) to explore the effects of polystyrene nanoparticles. Transcriptome analysis showed a wide genetic response. This study not only provides a new reference for understanding the mechanism of acute polystyrene nanoparticles-induced stress response, providing valuable ecotoxicological data for assessing the impact on invertebrate and vertebrate aquatic species.

Chen et al. described NHE and NKA gene families in the cobia fish (*R. canadum*) and their response to salinity changes. The results provide valuable insights into the molecular mechanisms governing ion transport and osmoregulation in *R. canadum*, contributing to developing strategies for enhancing aquaculture practices for this species.

Lin et al. studied the molecular cloning and expression profile of elongation of very long-chain fatty acids protein 6 (elovl6) in mud crabs (*Scylla paramamosain*) with dietary fatty acids, environmental salinity, and starvation stress. The findings of this study help understand the function and regulatory mechanism of fatty acid synthesis in crustaceans.

Liu et al. utilized single-cell transcriptome analysis to investigate the cellular immune response in dark sleeper fish (*Odontobutis potamophila*) infected with a co-pathogenic species *Aeromonas veronii*. The study contributes valuable insights into the immune response mechanisms in teleosts and provides a foundation for further research on cellular immunity in fish species.

The research conducted by Shu et al. investigated the impact of short-term water velocity stimulation on ovarian development in grass carp (*Ctenopharyngodon idellus*). The study provides valuable insights into the ovarian development of grass carp under short-term water velocity stimulation, offering potential regulatory genes and pathways for further ecological regulation strategies.

Zhang et al. explore the role of ionotropic glutamate receptors (iGluRs) in mediating excitatory neurosignals and environmental stress responses in Pacific oysters (*Crassostrea gigas*, Cg). Exposure to five heavy metals triggers a significant upregulation of *CgGRIA4* expression, indicating a robust response to metal stress. This research improves our understanding of iGluRs in metal stress response, signaling pathways, and environmental adaptability, paving the way for future investigations into cellular signaling mechanisms and neurotoxicity.

In conclusion, the diverse studies presented in this Research Topic collectively contribute to understanding physiological adaptations in aquatic organisms under various environmental stressors. From molecular pathways to physiological responses, these investigations offer valuable insights into the mechanisms governing organismal growth, endocrine regulation, and adaptation to environmental challenges in fish and mollusks. By elucidating the intricate interplay between genetics, environment, and physiology, these studies lay a foundation for further research on aquatic organism health and ecosystem sustainability.

Exploring the molecular and cellular mechanisms underlying endocrine regulation and physiological adaptations in response to environmental stresses is essential for safeguarding aquatic ecosystems. Further research should focus on integrating omics approaches, advanced imaging techniques, and environmental monitoring to deepen our understanding of how aquatic organisms cope with changing environmental conditions. By unraveling the complexities of adaptation mechanisms, we can better inform conservation efforts, sustainable aquaculture practices, and environmental management strategies to preserve the health and diversity of aquatic ecosystems for future generations.

